# Doctors as Appointed Fiduciaries: A Supplemental Model for Medical Decision-Making

**DOI:** 10.1017/S096318012100044X

**Published:** 2022-01

**Authors:** Ben Davies, Joshua Parker

**Affiliations:** 1 Uehiro Centre for Practical Ethics, University of Oxford, Oxford OX1 1PT, UK; 2 Department of Research and Education, Wythenshawe Hospital, Manchester M23 9LT, UK

**Keywords:** autonomy, medical decision-making, shared decision-making, doctor–patient relationship

## Abstract

How should we respond to patients who do not wish to take on the responsibility and burdens of making decisions about their own care? In this paper, we argue that existing models of decision-making in modern healthcare are ill-equipped to cope with such patients and should be supplemented by an “appointed fiduciary” model where decision-making authority is formally transferred to a medical professional. Healthcare decisions are often complex and for patients can come at time of vulnerability. While this does not undermine their capacity, it can be excessively burdensome. Most existing models of decision-making mandate that patients with capacity must retain ultimate responsibility for decisions. An appointed fiduciary model provides a formalized mechanism through which those few patients who wish to defer responsibility can hand over decision-making authority. By providing a formal structure for deferring to an appointed fiduciary, the confusions and risks of the informal transfers that can occur in practice are avoided. Finally, we note how appropriate governance and law can provide safeguards against risks to the welfare of patients and medical professionals.

## Introduction

According to a popular historical view of medical ethics, ultimate responsibility for healthcare decision-making has transferred over recent decades from doctors to patients.^1^ While this story is open to question,^2^ there has certainly been a shift away from paternalism over recent decades, and not without good reason. For instance, patients and doctors may have different values, and since a decision about my health is a decision about *me*, it seems important that my values are realized. Locating ultimate authority with patients may also help in avoiding some abuses of power by medical professionals.

Responsibility is often to be welcomed: the bearer of responsibility typically has authority and must be listened to. But it is also a burden, even when it is a welcome one.^3^ It is thus important to consider whether the burden is justified. That burden is rarely clearer than in certain healthcare decisions. Medical decisions are difficult, and patients may therefore worry about *getting them wrong.* Patients may anticipate that if their choices turn out badly, they will wonder what might have been if they had chosen differently and regret not making the right choice. In addition, the difficulties of medical decision-making may lead patients to feel overwhelmed even if they have no particular, identifiable concerns about how they or others will feel if things go wrong. Being confronted with a major decision can simply be overwhelming, even if there are no concrete consequences about which one has concern.^4^ Finally, being a patient is often unexpected, and disruptive. In some cases, depending on the severity of the illness, it intrudes significantly on one’s life and imposes significant burdens, such as severe pain, limitation of opportunities, or loss of control. The burdens of illness are bad enough but the added worries of being responsible for decision-making can seem unfair at a time of vulnerability when illness can undermine one’s capacity to exercise responsibility.

This paper does not advocate a purely paternalistic model. Rather, what we advocate is that patients should have the option to formally hand over decision-making authority to their doctor, who thus acts as an “appointed fiduciary.” This model of decision-making will not suit every patient and may be unsuitable for most. But for patients who sit between the two stools of lacking capacity on the one hand and being mentally and emotionally prepared to take on decision-making responsibility on the other, we believe that our proposal fills a gap not accounted for by other decision-making models in medical ethics. While in practice patients may ask doctors to make decisions in an informal way (“Oh I don’t know doctor, you just do what you think is best”), there is no formal mechanism for this. As such, our proposed model is novel, though not unprecedented.

Our goals in this paper are therefore twofold. Firstly, we want to outline in more detail how such a model might be compatible with patient autonomy. Some discussions of deferred decision-making see it as undermining of patient autonomy to a degree, but nonetheless endorse such a practice on the grounds that autonomy is not the only source of value. Our view is that this practice is more in line with patient autonomy than such a stance suggests. Secondly, much of the actual and theoretical approach to deferred decision-making is highly informal, and therefore open to potentially making things worse both for vulnerable patients and for doctors. Our proposal is to formalize this model, allowing for the deferral of decisions such that patients and medical professionals are guarded from harms.

## Doctors as Value Advisers

One model of the doctor–patient relationship assumed in much academic literature offers a neat division between facts—on which doctors are competent to offer their views—and values, which they are not. As such, one name for this model is the “informative model.”^5^ As Myfanwy Davies and Glyn Elwyn put it, this is a “radically individualized concept of patient autonomy.”^6^

This distinction may be a mistake. Julian Savulescu^7^ argues that if doctors are willing to engage noncoercively with their patients in a dialogue, it may be of great value for patients to hear their doctors’ views on the value of particular health decisions. On Savulescu’s view—“liberal rationalism”—patient and doctor discuss what is overall best. It is therefore a form of “shared decision-making,” which is increasingly prominent in medical ethics and practice.

The liberal rationalist model receives some support from the potentially burdensome nature of normative decision-making. Open discussion between doctor and patient may help the doctor to understand their patient better; doctors’ medical knowledge means they can combine relevant empirical knowledge with value recommendations in a way that others (such as family) cannot; and, as Savulescu points out, doctors typically also have considerable experience of how people are affected by illness, not only in a medical but also an emotional and psychological way. So long as a doctor offering value-based as well as fact-based advice will make the decision less burdensome for the patient, this provides an additional reason to do so.

However, things are complicated for the liberal rationalist when we recognize that even reasonable, rational discussion can have the effect of increasing a person’s decision-making burden. A patient who enters a doctor’s surgery with a clear sense of what they want may have their burden increased if a doctor challenges their values. It is important to acknowledge that the relationship between rational discussion and the burdens associated with responsibility for decision-making is not always positive. Doctors following this model may sometimes have to make a judgment call about the balance between the value of patients being more informed about relevant values, and the burdens associated with being unable to process such information.^8^

## Appointed Fiduciaries

Liberal rationalism mandates that health decisions be arrived at following rational discussion between doctor and patient. Savulescu makes an important distinction between the values that it would be best to live by, and the values that it is legitimate to *compel* people to live by. The liberal rationalist proposal aims to expose patients to reasoned argument in the hope of getting them to make better decisions but does not sanction compulsion. Ultimately, the decision is still the patient’s.

However, a recognition of the potentially burdensome nature of responsibility for one’s own health decisions, particularly for those in very poor health, might take us further, though still not all the way to paternalistic compulsion. Liberal rationalism marks a point between the paternalistic “doctor knows best” model, and the model of the patient as an isolated, autonomous individual. A different point in this region, a little further toward the paternalistic model, but which does not require a return to the autonomy-undermining elements of that model, is the idea of the doctor as appointed fiduciary, where the doctor acts as a patient’s representative, nominated to make important decisions for them, in their interests.

As Zara Bending points out, a simple fiduciary model (e.g., one where doctors are assumed as a default to take on this role) is “undesirable as it places little if no emphasis on the right of an autonomous individual to make decisions regarding his or her health.” Indeed, she suggests that the model is “paradoxically flawed insofar as it cannot promote the autonomous rights of patients within a fundamentally paternalistic paradigm.”^9^ With respect to a general model of the doctor–patient relationship, Bending is quite right. If doctors are assumed to be patients’ fiduciaries automatically, there seems to be no room for patient autonomy. However, the fiduciary model would be implemented neither automatically nor in all cases. We can adopt the idea of the doctor as acting on behalf of the patient, but only when requested by the patient.

The idea of doctors as a fiduciary is acknowledged, albeit implicitly, by some health care guidance. For instance, the General Medical Council advises doctors that if a patient “asks you to make decisions on their behalf…you should explain that it is still important that they understand the options open to them, and what the treatment will involve.” Although the guidance does not explicitly endorse such deferral, its failure to rule it out suggests an implicit endorsement. But the idea of patients with capacity deferring decisions to their doctor seems to be treated as a problem to be avoided if possible, and managed if not. In addition, the guidance suggests that “No one else can make a decision on behalf of an adult who has capacity.”^10^ Yet the appointed fiduciary model endorses precisely this.

Furthermore, the guidance reflects the reality of medicine in that such a transfer of decision-making authority is ultimately conducted in an informal way. Although patients may feel reassured if a doctor informally agrees to take on decision-making, we worry that such informality has three potential downsides. First, it may leave the patient misinformed about who retains ultimate decision-making authority. Second, it is more open to error, where doctors are mistaken about a patient’s preferences around decision-making, than a more formal transfer of authority. For instance, a doctor who has offered two choices to a patient, and expressed an opinion about which is preferable, might be told “You choose.” This could be interpreted as a patient agreeing with and taking responsibility for the option that the doctor has promoted, *or* as a patient asking the doctor to take on decision-making responsibility. Finally, the informal approach sets the context of a patient transferring decision-making authority as something the patient must *pursue*, rather than an option that is offered to them.^11^ Yet as we discuss below, this risks leaving vulnerable patients in the position of wanting to transfer responsibility, but unsure whether they can do so, or unconfident in how to approach their doctor.

On the appointed fiduciary model, patients will still typically be fully in charge of their own health care decisions, with doctors providing (at least) empirical information about treatment options, as well as, perhaps, normative opinion along the liberal rationalist line. However, what distinguishes the appointed fiduciary model is that patients have the option of taking this recourse to medical expertise beyond the level of consultation, and entrusting doctors with decision-making power in a formal, legal transfer of trust.

We have outlined how this model differs from the current, informal way that decision-making might be transferred. The appointed fiduciary model also differs from traditional paternalism in several ways. The default assumption is that the patient makes the ultimate decisions about their treatment; doctors only acquire this power if it is transferred to them by the patient. Clearly, there is room for disagreement about the status of such autonomous decisions to give up one’s autonomy. In part, this depends on what we think the value of autonomy consists in. One view is that autonomy is valuable in a synchronic sense, as an expression of an individual’s current values. On this view, it seems clear that we should respect an individual’s autonomous decision to give up future autonomy. On the other hand, we might think that autonomy’s value is diachronic (i.e., that it should be protected or even maximized over the course of one’s life), so that a decision that severely threatens one’s future autonomy should not be respected by others, even if that decision is itself autonomous. Examples of such decisions might include the decision to sign oneself into indentured servitude, or to undergo a medically unnecessary lobotomy that would remove crucial reasoning capabilities. Yet the appointed fiduciary model does not threaten the individual’s *capacity* for autonomy.

A second difference from standard paternalism is that any transfer of decision-making authority is ultimately open to challenge by the patient. It may be helpful to distinguish between two senses of control at this point. The standard, informational model of medical decision-making involves patients taking “active” control over medical decisions. It is the patient who takes each decision, guided and aided by the doctor. Paternalism offers no control over decision-making. The sense of control at work in an appointed fiduciary relationship, in contrast, is “conditional” control. Although the patient does not actively guide the decision-making process, he retains the option of removing the doctor’s decision-making authority at any stage.

The model also differs in a third way by virtue of the standard transfer of decision-making authority being far more local. Allen Buchanan and Dan Brock invoke a fine-grained notion of capacity when deciding whether a patient with cognitive limitations should be able to decide for themselves.^12^ That a patient lacks capacity to make one kind of decision (whether they should take their medication) does not mean that they lack capacity to make a different decision, for example, where the stakes are lower, or where different rational skills are required. Similarly, appointing one’s doctor as fiduciary with respect to a particular medical decision does not have any necessary impact on the patient’s right, internal capacity or external opportunity to decide on a host of other decisions.

A fourth, final departure from paternalism is that the appointed fiduciary model is compatible with the claim that the fiduciary has obligations, in line with the liberal rationalist model, to understand their patient’s values and priorities. Unlike the original liberal rationalist model, the emphasis may be less on getting patients to understand *why* a particular course of action is best for them, and more on the doctor acquiring relevant information that helps them make that decision. Additionally, one might argue that if patients are to genuinely retain the option of taking back decision-making control at some stage, they must be kept abreast of at least a key minimum level of information. Whether this latter consideration is decisive is beyond the scope of this paper; what is important is to note that whether, and to what extent, patients must be kept informed about their treatment and condition is a distinct question from the issue of decision-making authority.

### Shared versus Split Decision-Making

A theme of increasing prominence in discussions of the doctor–patient relationship is the idea of “shared decision-making,” described by the United Kingdom’s National Institute for Health and Care Excellence (NICE) as decisions where “health professionals and patients work together.”^13^ As Cathy Charles et al. note, the details of shared decision-making are not always made entirely clear by proponents.^14^ Nevertheless, Ezekiel and Linda Emmanuel propose two models in between absolute patient sovereignty and classical paternalism.^15^ According to the “interpretive” model, the doctor helps the patient to work out the patient’s own values; on the deliberative model, of which liberal rationalism is an instance, the doctor aims to persuade the patient to adopt her favored course of action, though the decision is ultimately left up to the patient. For some, shared decision-making involves some hybrid of interpretative and deliberative models.^16^ Although patients are not left without support, on all these views of shared decision-making the ultimate decision must be the patient’s, not someone else’s.

Finally, Davies and Elwyn describe shared decision-making as a model which involves “describing relevant options and supporting patients’ decisions to the extent that they seek to exercise choice.”^17^ Similarly, the guide by England’s National Health Service (NHS) on shared decision-making suggests that “Individuals should be supported to play as active a role as they wish in decisions about their care.”^18^ Of course, if the default mode of interaction is that of information provision, patients may not be aware that they can take a less active role in medical decision-making, and it may be left to the doctor to spot when a patient seems overwhelmed. Davies and Elwyn suggest instead that we should “prefer an optional autonomy approach.” This idea is left somewhat underspecified. There are risks in leaving it entirely up to patients to “seek” to exercise choice, or to demonstrate a precise level of “active” involvement in their care. Medical settings can be intimidating to all of us and many patients place great trust in doctors; if we leave things entirely up to the patient there is a danger of doctors misreading caution, uncertainty or nervousness as passivity. While this might get the right result for the patients with whom we are principally concerned (i.e., those who have capacity but would prefer someone else to make decisions), it may get the wrong result for another set of patients: those who are overwhelmed or intimidated by a medical setting, but who would ultimately prefer a significant role in decision-making and therefore need support, not a fiduciary. Davies and Elwyn do suggest that an optional autonomy approach requires that we ascertain how much of a decision-making role the patient wants. Importantly, then, this may not require the patient to take all the initiative in “seeking” autonomy. Nonetheless, unless we have full autonomy as a default, overridden only by an explicit request from the patient, we risk misinterpretation of patients’ wishes, and overwhelmed patients failing to express their desire for greater autonomy.

Although the NHS England and Davies–Elwyn proposals are clearly of value, it is helpful to distinguish between shared decision-making and what we call “split” decision-making. In the appointed fiduciary model, the patient makes the initial decision whether to transfer authority, perhaps guided or assisted by his doctor or others. The doctor then takes up the decision-making authority, acting according to what she deems best for the patient, with as much input from the patient as the patient desires. Although the patient retains the option of reclaiming control, the separateness of this decision means that this is not ultimately a form of shared decision-making. Even if shared decision-making should be the default of medical practice, this does not mean that it is appropriate in every context. Furthermore, since the appointed fiduciary model is the subject of a concrete *offer* or *question* to the patient, it allows the patient to shape their role in their own care without presuming that a patient who wants to be actively involved will get actively involved.

In our view, then, many cases of shared decision-making are either underspecified with respect to the central problem that concerns us, that is, patients who, while retaining decision-making capacity are excessively burdened by medical decision-making, or simply do not go far enough because they still leave the patient with ultimate decision-making authority in all cases. Paternalist models and, to a lesser extent, “optional autonomy” models risk going too far the other way, and depriving patients who would like to exercise autonomy of realistic opportunities to do so. We therefore suggest that the appointed fiduciary model represents an important *option* for at least some patients, though we do not endorse it as a generic approach to medical decision-making.

## Objections

### Patient Preferences

One might object to the appointed fiduciary model on several grounds. The first is that patients simply do not want such a model. Patients, one might argue, who are sufficiently competent to make certain decisions will not want to hand over their decision-making power.

Only a very strong version of this claim—that all or almost all patients have no desire to hand transfer decision-making—would challenge our argument. Since the model is neither mandatory nor universal, the fact that most patients will not adopt it is not in itself an argument against it. In any case, it is doubtful that competent patients really do uniformly want to have decision-making power in all medical issues. Charles et al. note that “research has shown… that while patients typically express high preferences for information about their illness and its treatment… their preferences for participation in treatment decision-making are much more diversely distributed,”^19^ and Bullock cites several studies suggesting that “patients become increasingly averse to making medical decisions the sicker they are.”^20^ Similarly, Neeraj Arora and Colleen McHorney found that 69% of just over 2,000 patients in the United States with chronic conditions either agreed or strongly agreed with the statement “I prefer to leave decisions about my medical care up to my doctor;” the responses show several patterns, with older, less educated, and more severely ill patients all more likely to want a passive role.^21^ A much larger meta-analysis found a majority in favor of shared decision-making, but a still sizeable minority (32%) preferring that their doctor make decisions.^22^ Finally, Jack Ende et al. find “no correlation between patients’ decision-making and information-seeking preferences,” suggesting that patients’ desires to understand what is happening to them does not necessarily translate to a desire to actually make decisions.^23^

### Patient Welfare

The appointed fiduciary model is primarily motivated by the worry that some patients who are capable of exercising autonomy may find it excessively burdensome to do so with respect to certain healthcare decisions. One worry about this proposal is that, by retaining ultimate control for the patient over treatment options, we do not actually solve this problem at all. If what is burdensome for the patient is the idea that he is ultimately at the reins, appointing a fiduciary may seem to retain this feature, since this aspect of the relationship can be terminated, and it ultimately remains the patient’s decision whether to do so.

However, while this ultimate oversight might be a cause of stress to some patients, this worry shows an incomplete understanding of the burden of responsibility. One burdensome element of being responsible is the taking of individual decisions, large and small, where there is a risk of choosing poorly. Another is the need to consider a multiplicity of factors in trying to decide what is best, at the same time as managing other burdens such as physical pain, increased stress in relationships, a reduced capacity to do one’s job, and so on. When all available options are emotionally difficult to confront, emotional conflict can occur, resulting in decision paralysis.^24^ The appointed fiduciary model removes these sources of stress. Even though the patient both retains the right to take charge and is responsible for the original decision to appoint a fiduciary, the number of decisions for which they are responsible in an active way, and the emotional intensity of those decisions, may be decreased.

In addition, the way in which one is responsible for appointing one’s doctor as a fiduciary, while it remains a form of responsibility, is importantly different than responsibility for treatment decisions. The appointment of a doctor (suitably guided by conversation with oneself) is the appointment of an expert who is themselves accredited by a body of professional standards. Experts certainly make mistakes, and their reasonable decisions may turn out not to be overall for the best. This means that it will sometimes turn out to have been the wrong choice to trust an expert. But so long as you follow an expert’s advice sincerely, it would be unreasonable to accuse you of acting irresponsibly, or to hold you responsible for their mistakes or erroneous decisions.

### Giving Up Autonomy

A further worry might be that handing over the responsibility for important decisions may express or comprise worrying attitudes about the patient as an autonomous agent, even if it does not directly undermine his autonomy. Tim Scanlon suggests that “In a situation in which people are normally expected to make choices of a certain sort for themselves … not having or not exercising this opportunity would be seen as reflecting a judgment (their own or someone else’s) that they are not competent or do not have the standing normally accorded an adult member of the society.”^25^

While this is clearly true of some cases, it does not seem true as a general rule. For instance, competent adults are normally expected to make their own selection from a restaurant menu. But there seems to be no necessary judgment—on my part or anyone else’s—if on one occasion I simply cannot choose and ask someone else to do so for me. While some patients may want to appoint their doctor as fiduciary because they judge themselves to be insufficiently competent or inferior in some other way, others may do so precisely because, although they know that they have the rational faculties and information to make a decision, they do not *want* to do so. Such a judgment does not require that one thinks of oneself as incompetent, nor does it requires that others think of you this way. And although there is in one sense a judgment of inferiority—the patient deems themselves to be less capable to make the relevant decision *in this instance* than their doctor—this is not essentially related to a patient’s standing, but to their emotional proximity to the decision.

An alternative concern might take the form articulated by Robert Paul Wolff.^26^ Wolff argues that, as autonomous individuals, we have an obligation not to do things just because an authority figure has told us to. Wolff uses this as the basis of an argument against legitimate political authority, but it might also be read as an argument for a certain model of expertise: one where we can take expert advice but must ultimately make all decisions for ourselves. On this view, then, the appointed fiduciary model does not represent a moral failure of the doctor, but of the patient. Similarly, Madder argues that “The threat to patient autonomy lies in the patient’s responsibility for whole-life decisions. Responsibility for oneself is in my view central to autonomy. By taking responsibility for decisions which affect our lives, we maintain our discreteness as self and enable self-realization.”^27^

The modern world is complex, and although we may have an obligation to understand certain issues, and to think for ourselves when it comes to making decisions about them, this is less plausible when it comes to decisions that predominantly affect ourselves. Although a person’s medical decisions do affect others around them, the core idea of patient sovereignty is that it is ultimately the patient’s decision to make. Since the appointed fiduciary model is voluntary, a patient will ideally only adopt it when they trust their doctor, and genuinely feel that it will be too burdensome to make the decision themselves (though see below). One might well worry if a competent individual deferred *all* or *most* important decisions. But it is not clear why the model should be any more problematic than other important decisions (such as legal or financial decisions) for which fiduciaries can be appointed.

Relatedly, one might locate the problem in the moral relationship that is generated by the appointed fiduciary model. In particular, the mode involves the medical professional taking on responsibility on behalf of the patient, at least in a limited context. Richard McMillan draws a distinction between taking responsibility “for” someone, and being responsible “to” them, claiming that the former necessarily requires that we “manipulate or control their behavior to achieve the outcome we desire.” He argues, further, that this essential involves the “denial of the patient’s moral agency.”^28^

However, on McMillan’s taxonomy, we may frame the appointed fiduciary model as a form of responsibility “to,” rather than “for.” The patient retains ultimate control, and the doctor’s responsibility is conditional on the patient accepting her recommendations. As such, it may be most accurate to describe the model as a form of joint responsibility, where the doctor is responsible for relevant health outcomes *conditional on* the patient following guidelines. The responsibility transferred is a level of *decision-making* responsibility rather than sole, ultimate responsibility for the medical outcome.

Alternatively, we might reject McMillan’s straightforward distinction, where we are all responsible only for our own actions (and their impact on others), and each moral agent is responsible for themselves. Even if “responsibility for” does involve some sacrifice of moral agency, it is not clear that this is ethically problematic if it occurs in a limited context. Moreover, we can meaningfully speak of a doctor taking on responsibility *for* her patient in a limited sense because the patient has (autonomously) given over this responsibility. In this case, neither coercion nor manipulation seems required.

### Vulnerable Patients

A final patient-centered worry presents a challenge to this last claim. We suggested that worries about patients failing to take active control over medical decisions are misplaced, because the patient must at least take active control over the decision to take only conditional control over those decisions.

However, one might worry that this assumes a level of sophistication that is not present in all patients, and opens some vulnerable patients up to various forms of mistreatment. Even most doctors want the best for their patients, (1) some doctors do commit malpractice, and (2) there is some evidence of various biases^29^ in medical decision-making, including racial bias^30^; disability and mental health-based bias^31^; age bias; and gender bias.^32^ Whether these biases are conscious or implicit, it seems that in some cases, doctors will make a decision that is not in their patient’s best interests, apparently on the basis of demographic characteristics. To be clear, the concern is not that patients in these groups, merely by falling within a group which experiences bias, are inherently less capable of making decisions about their own healthcare. Within each group, there will be a range of capability. Rather, the concern is that there will be members of such groups who *are* vulnerable to being pushed unwillingly or uncomprehendingly into the appointed fiduciary model. For these patients, there is then a separate risk that existing biases will mean that their fiduciary is more likely to make decisions that are not in their best interests.

The fact that such biases already occur means that adopting an appointed fiduciary model will not introduce biases. Rather, the worry is that merely having the option of such a relationship may lead some doctors to push vulnerable patients to agreeing to such a model. This is already problematic, since it implies that the decision to adopt the model is not genuinely autonomous. Moreover, if a patient is pressured into accepting less influence over decisions affecting their own health by doctors who also have biases against that patient, this makes it more likely that the biases will negatively affect health. So, although the appointed fiduciary model does not introduce new biases, it may exacerbate the effects of existing ones.

This is no small problem.^33^ However, we can mitigate its force to some degree, with several considerations. Firstly, the problem of bias itself is to some degree independent of a specific doctor–patient relationship. As such, it is incumbent on a healthcare system to address this problem anyway. Even if it is a decisive objection to implementing this model *now* that it will make worse an already serious problem, this does not undermine its potential future implementation.

Secondly, the scope for both intentional abuse and unintentional overreach is at its greatest if the decision about whether to engage in an appointed fiduciary relationship is taken by the doctor and patient alone. However, this is not a requirement of the appointed fiduciary model. It is entirely compatible with this model that the decision to create a fiduciary relationship should be subject to external oversight, approval and review. Oversight and approval might come internally in the form of a second opinion, an ethics board, or a senior colleague, or externally by involving a patient’s friend or family member. Review—which might come as part of internal morbidity and mortality reviews—is less useful for protecting the individual patient but may act as a brake on doctors adopting the model rashly.

Thus, although the doctor adopts an important position of power over their patient when appointed as a fiduciary, that appointment itself is not solely overseen by that doctor, and she or he is not thereby left as a lone authority in the patient’s care. To be authorized to make decisions on someone else’s behalf need not mean that one has excessive power over them, not even in a limited part of their life.

### Doctors’ Welfare

Finally, it is important to note that while patients may be the primary subject of worries about vulnerability, doctors can be vulnerable too. Whereas patients may feel bound to responsibility for their own health by a role that has been unwelcomely thrust upon them, doctors’ responsibility is ultimately to be found in a choice to enter the medical profession. But the fact that responsibility is in some sense chosen does not make it easy. So, one might worry that even if this proposal makes the lives of some patients easier, it will do so at the cost of making doctors’ lives harder.

Still, although the appointed fiduciary role is potentially burdensome in several ways, it does not seem to introduce any novel burdens. Doctors will already often have some sense of what course of treatment is preferable for a particular patient, and are already often required to make best-interests decisions for patients who lack autonomy. Additionally, doctors have formal avenues by which they can share the burden of responsibility, such as discussions with colleagues or through the multidisciplinary team.

Perhaps more pertinent is the concern that the role of appointed fiduciary may expand doctors’ legal liabilities without also extending their legal protections. Much depends on the precise legal framework that exists around the role of fiduciary. The framework of oversight, approval and review outlined above may also provide some corresponding protection for doctors. A further possible protection is a requirement to record the justification (both clinical and based on assessment of the patient’s values) of specific decisions. It is important to remember that the appointed fiduciary relationship is ultimately a choice made by patients to place trust in their doctor. Although this does not give the doctor license to make just any decision, it also means that a patient cannot pursue legal action based solely on the fact that they are unhappy with their treatment outcome.

## Conclusion

We have outlined an extension to the doctor–patient relationship that aims to respond to the possibility that some patients will feel overwhelmed by the responsibility involved in making treatment decisions. While this extended model—appointing one’s doctor as a fiduciary—may not be appropriate for most patients, it provides an option for patients who fall between the two camps of having insufficient capacity to be responsible, and having both capacity and sufficient emotional/psychological resources to feel confident making a decision about their medical care. Since the appointed fiduciary relationship relies on patient choice to be activated, we have argued that it still sufficiently respects patient autonomy. We have also argued that, although there are legitimate concerns about both patient and doctor welfare arising from this model, an appropriate governance and legal framework can be developed to minimize both sets of concerns.

## Notes

1. General Medical Council. *Consent: Patients and Doctors Making Decisions Together.* Manchester: General Medical Council; 2008:e1–62, at e7–8; Rubin MA . The collaborative autonomy model of medical decision-making. Neurocritical Care 2014;20:311–8.2423381410.1007/s12028-013-9922-2

2.

McCullough LB . Was bioethics founded on historical and conceptual mistakes about medical paternalism? Bioethics 2011;25:66–74.2117570910.1111/j.1467-8519.2010.01867.x

3.

Kapp MB . Health care decision making by the elderly: I get by with a little help from my family. The Gerontologist 2001;31:619–23, at 620.10.1093/geront/31.5.6191778487

4.

Dawson A. The future of bioethics: Three dogmas and a cup of hemlock. Bioethics 2010;25:218–25, at 221.10.1111/j.1467-8519.2010.01814.x20500759

5.

Emmanuel EJ , Emmanuel LL . Four models of the physician–patient relationship. The Journal of the American Medical Association 1992;267:2221–6, at 2221.1556799

6.

Davies M , Elwyn G. Advocating mandatory patient ‘autonomy’ in healthcare: Adverse reactions and side effects. Health Care Analysis 2008;16:315–28.1797572910.1007/s10728-007-0075-3

7.

Savulescu J. Rational non-interventional paternalism: Why doctors ought to make judgments of what is best for their patients. Journal of Medical Ethics 1995;21:327–31877845510.1136/jme.21.6.327PMC1376828; Savulescu J. Liberal rationalism and medical decision-making. *Bioethics* 1997;11:115–29. See also Savulescu J, Momeyer RW. Should informed consent be based on rational beliefs? *Journal of Medical Ethics* 1997;23:282–8.

8. For example, Bullock EC . Mandatory disclosure and medical paternalism. Ethical Theory and Moral Practice 2016;19:409–24. This more widely discussed issue, sometimes described as a “right not to know,” (e.g., Takala T. Genetic ignorance and reasonable paternalism. *Theoretical Medicine* 2001;22:485–91; Andorno R. The right not to know: An autonomy based approach. *Journal of Medical Ethics* 2004;30:435–40; Bortolotti L. The relative importance of undesirable truths. *Medicine, Health Care and Philosophy* 2013;16:683–90) is orthogonal to the issue of deferred decision-making. However, if patients do have such a right, this adds attractiveness to the appointed fiduciary model; it would be entirely understandable for a patient who did not wish to know medically relevant information himself to conclude that since his doctor is better informed than he is, she should ultimately take charge of decision-making. We do not take a position on this here, but it is worth noting that some medical interventions will require patients to receive some information, for example, about when and in what circumstances to take medication; which symptoms might indicate a need to return to hospital.

9.

Bending ZJ . Reconceptualising the doctor–patient relationship: Recognising the role of trust in contemporary health care. Bioethical Enquiry 2000;12:189–202, at 190.10.1007/s11673-014-9570-z25124984

10. General Medical Council. *Consent: Patients and Doctors Making Decisions Together.* Manchester: General Medical Council; 2008:e1–62, at e11.

11.

Sinding C , Hudak P , Wiernikowski J , Aronson J , Miller P , Gould J , Fitzpatrick-Lewis D. “I like to be an informed person but…” Negotiating responsibility for treatment decisions in cancer care. Social Science and Medicine 2010;71:1094–101, at 1097.2063397010.1016/j.socscimed.2010.06.005

12.

Buchanan A , Brock D. Deciding for Others: The Ethics of Surrogate Decision-Making. Cambridge: Cambridge University Press; 1989.

13. National Institute for Health and Care Excellence. *Shared Decision Making Collaborative: A Consensus Statement*; available at https://www.nice.org.uk/Media/Default/About/what-we-do/SDM-consensus-statement.pdf (last accessed 4 Nov 2019).

14. Charles C, Gafni A, Whelan T. Shared decision-making in the medical encounter: What does it mean? (or it takes at least two to tango). *Social Science & Medicine* 1997;44:681–92. See also Lewis J. Getting obligations right: Autonomy and shared decision-making. *Journal of Applied Philosophy* 2020;37:118–40. doi:10.1111/japp.12383.

15.

Emmanuel EJ , Emmanuel LL . Four models of the physician–patient relationship. The Journal of the American Medical Association 1992;267:2221–6.1556799

16.

Frosch DL , Kaplan RM . Shared decision making in clinical medicine: Past research and future directions. American Journal of Preventive Medicine 1999;17:285–941060619710.1016/s0749-3797(99)00097-5; Lisska EE. Do patients want to participate in medical decision-making? A critical review of the literature. *Yale Medicine Thesis Digital Library*; 2004.

17.

Davies M , Elwyn G. Advocating mandatory patient ‘autonomy’ in healthcare: Adverse reactions and side effects. Health Care Analysis 2008;16:315–28, at 318.1797572910.1007/s10728-007-0075-3

18. NHS England. *Shared Decision Making: Summary Guide.* London: NHS England; 2019:e10.

19. Charles C, Gafni A, Whelan T. Shared decision-making in the medical encounter: What does it mean? (or it takes at least two to tango). *Social Science & Medicine* 1997;44:681–92, at 683.

20.

Bullock EC . Mandatory disclosure and medical paternalism. Ethical Theory and Moral Practice 2016;19:409–24, at 410.

21.

Arora NK , McHorney CA . Patient preferences for medical decision making: Who really wants to participate? Medical Care 2000;38:335–41.1071835810.1097/00005650-200003000-00010

22. Lisska EE. Do patients want to participate in medical decision-making? A critical review of the literature. *Yale Medicine Thesis Digital Library*; 2004.

23.

Ende J , Kazis L , Ash A , Moskowitz MA . Measuring patients’ desire for autonomy: Decision making and information-seeking preferences among medical patients. Journal of General Internal Medicine 1989;4:23–30, at 23 264440710.1007/BF02596485. See also Winzelberg GS, Hanson LC, Tulsky JA. Beyond autonomy: Diversifying end-of-life decision-making approaches to serve patients and families. *Journal of the American Geriatrics Society* 2005;53:1046–50.

24.

Gawande A. Complications: A Surgeon’s Notes on an Imperfect Science. London: Profile Books; 2002 ; Botti S, Iyengar SS. The dark side of choice: When choice impairs social welfare. *Journal of Public Policy and Marketing* 2006;25:24–38.

25.

Scanlon T. What We Owe to Each Other. London: Belknap Press; 1998:253.

26.

Wolff RP . In defense of anarchism. Journal of Philosophy 1971;68:561–7.

27.

Madder H. Existential autonomy: Why patients should make their own choices. Journal of Medical Ethics 1997;23:221–25, at 221–222.927974310.1136/jme.23.4.221PMC1377270

28.

McMillan RC . Responsibility to or for in the physician–patient relationship? Journal of Medical Ethics 1995;21:112–115.760893410.1136/jme.21.2.112PMC1376634

29.

Fitzgerald C , Hurst S. Implicit bias in healthcare professionals: A systematic review. BMC Medical Ethics 2017;18:e1–18.2824959610.1186/s12910-017-0179-8PMC5333436

30.

Green AR , Carney DR , Pallin DJ , Ngo LH , Raymond KL , Iezzoni LI , Banaji MR . Implicit bias among physicians and its prediction of thrombolysis decisions for black and white patients. Journal of General Internal Medicine 2007;22:1231–81759412910.1007/s11606-007-0258-5PMC2219763; Chapman EN, Kaatz A, Carnes M. Physicians and implicit bias: How doctors may unwittingly perpetuate health care disparities. *Journal of General Internal Medicine* 2013;28:1504–10.

31. British Medical Association. *Recognising the Importance of Physical Health in Mental Health and Intellectual Disability: Achieving Parity of Outcomes.* London: British Medical Association; 2014:72.

32.

Rogers W , Ballantyne A. Gender and trust in medicine: Vulnerabilities, abuses and remedies. International Journal of Feminist Approaches to Bioethics 2008;1:48–66.

33.

Beauchamp T , Childress J. Principles of Biomedical Ethics. 5th ed. Oxford: Oxford University Press; 2001:93 cites the potential for abuse as an argument against any “general practice of allowing waivers” of rights. However, they recognize that individual waivers may be permissible, subject to “appropriate procedural responses.”

